# Rabies1Read before the Iowa-Nebraska State Veterinary Medical Association, October 1, 1902.

**Published:** 1902-11

**Authors:** A. J. Pistor

**Affiliations:** Fremont, Neb.


### RABIES.[1]

By A. J. Pistor, D.V.S.,

FREMONT, NEB.

As our knowledge of diseases and medicine increases so does the subject of preventive medicine take a more important place in the medical profession. When considering a medical problem to-day one of the important factors considered is that of prevention, and in the case of infectious diseases that also of eradication. In this the quarantine laws and the methods of isolation become factors of great importance; and that they shall be of value they must be constructed in such a manner that they will conform with our advanced ideas.

From a standpoint of practice rabies must truly be considered as to its methods of prevention. With this idea in view I will briefly go into a description of our knowledge of rabies, and will then consider rabies from a standpoint of prevention, and with this would naturally follow its eradication.

Rabies is an acute specific disease caused by a specific organized substance. It is propagated by direct inoculation either naturally or artificially. By natural inoculation I mean either by the bite of a rabid animal or by the virulent matter being absorbed into a wound or abraded skin, as would happen when a rabid animal is

[1] Read before the Iowa-Nebraska State Veterinary Medical Association, October 1, 1902.

allowed to lick the skin. Artificial inoculation relates to laboratory methods.

That there is a specific agent which causes the disease has been proven by every investigator who has had any impartial dealings with the disease. He has inoculated and reinoculated the virulent matter, with practically the same results in every case; and when a sufficient number of parallel test inoculations were made the experiments were continued from the original virulent matter through a long series of animals. The active virus has not as yet been described, yet the burden of proof all points toward an organized body. Paul Bert proved that virulent saliva from rabid dogs could be made inactive by passing the saliva through a plaster filter, thereby arguing that the virulent part must have some structure or it would not be capable of being detained in the pores of the filter. Its history, its mode of propagation, its period of incubation, its pathological lesions, all indicate an organic virulent agent. Australia has remained free of the disease, because the active agent was excluded from the country by a strict quarantine regulation of all imported dogs. England has now rid itself of rabies by strict measures, and only through the introduction of a new case from some other country will it again appear in that country. The infectious substance is produced or grown in the living body, and soon loses its virulence after the death of the body which harbors it.

Children and animals which practically are not capable of worry are the victims in the greatest number of cases. This would be a way to overcome the psychological cause, as argued in the past, but which argument is hardly necessary in view of the large amount of positive evidence reported in every outbreak and proven in every set of experiments as to the actual existence of a virus.

Rabies is reported in many species of animals. Even birds (pigeons) are not immune, as reported by Kraus and Clairmont. Its greatest numbers of victims are among the dogs; but cattle, horses, pigs, and other domestic animals are also frequently affected. The age, sex, or condition of an animal is no guard against its invasion. From every clime come the reports of its existence. In this section of the country it is greatly on the increase, and consequently it is of great interest to the economists of this district.

Its period of incubation varies greatly, and no positive opinion can be given as to its length of time in some individual or animal after the same has been bitten. It lasts from a very few days to a year, or even longer, some cases being reported where the period lasted fourteen months before the symptoms manifested themselves. Pasteur has proven that the smaller the amount of virus injected the longer the period of incubation. Again, the nearer the nervous centre the inoculation was made the shorter the period. The less contaminated the virus the shorter the period of incubation; so virus taken from the brain proved to be the most active in its results.

The symptoms are quite similar in all species, and are mainly of a nervous order, mental excitement being the most prominent manifestation. A change in the demeanor of the animal will first be noticeable. The animal may be a least bit uneasy, and will seek some quiet or dark place, but will even move about restlessly while there. It will try to crawl under some straw or rags or anything around ; then it will tear and shake these articles, and will swallow some of them. It will howl, and its howl will be strained and unnatural. It may be more friendly or more vicious than usual; then it will attempt to run away, and if loose will run, again returning or dying on the road. It will bite at all objects that may happen in its path. Horses will smash their stalls, rub their lips so that they become lacerated, kick, bite, and be generally vicious; sometimes they act colicky. Cows will bellow, run, suddenly stop, throw about their heads, and snort, chase, and try to hook other animals. Hogs will tear up the bedding, bite at articles with which they come in contact; also bite at animals. Gradually paralysis sets in, and then death ends their suffering.

Clinically there are two forms of the disease—the furious or violent and the dumb or sullen. Between the two there are all stages of modification. In the dumb form the vicious period is usually absent or hardly noticeable, the paralytic stage immediately following the initial or melancholic stage.

The symptoms usually last from three to five days, but may be as short as two days or as long as ten days. In birds it lasts longer than this, and the bird virtually dies of starvation, due to paralysis of the larynx and pharynx.

The sections are diagnostic by their negative presentations. Besides some inflammatory lesions on the mucous membranes there are few other manifestations. The stomach of the dog may and usually does contain foreign matter. The bowels are quite empty. Some signs of constipation may be present. The microscopical lesions are of such a nature as to be of little use to the general practitioner, hence I will omit them.

Having briefly and hurriedly gone over the pathological discussion of rabies, I have omitted many points which would be necessary to make that subject complete; but I think that I have enumerated the most important points, and will now finish my subject with the treatment and prophylaxis of rabies.

When a person or an animal is bitten by a rabid animal it is first in line to rid the wound of any virus which may have been inoculated, or else to so destroy the virus that it loses its virulence. Nothing being at hand, sucking the wound very forcibly would be in order. This would have a tendency to draw out the virus, and would cause a greater flow of blood, thereby washing out the virus and avoiding absorption ; or excision of the wounded parts may be practised. The actual or chemical cautery may be applied, as the hot iron, corrosive sublimate, nitrate of silver. Antiseptics, as creolin, carbolic acid, etc., may be used as a dressing. The foregoing must be applied as soon as possible after the injury. If too long a time is allowed to elapse the virus may be absorbed, and then local treatment will be of no avail.

The Pasteur treatment is next in line where human subjects have been inoculated or bitten by a rabid animal. This treatment has been extensively administered, and statistics are very much in its favor. After the symptoms appear no treatment has proven itself efficacious.

When a person is bitten it is advisable, if possible, to secure the attacking animal, place it where it can do no further harm, and then carefully observe the developments. If there is any doubt about the case a part of the brain may be inoculated into another animal or two. Rabbits answer for this purpose very nicely. By inoculating these test animals into the coats of the brain results are more readily and positively obtained, a rabbit reacting in from eleven to twenty-one days.

To introduce preventive measures is no easy matter. On this score one encounters many obstacles. If one could be assured that the preventive measures would be obeyed to the letter the stamping-out process would become a simple matter. In this case dogs are our first object of attack, because the disease most often appears in them and because it is spread by direct inoculation—a bite. Dogs are allowed the freedom of our cities and country, and thereby the spread of rabies is greatly facilitated.

To inaugurate any measures which will control these dogs would immediately arouse the antagonism of a large percentage of dog owners. They would personally agitate against such measures, and under the cloak of humane societies they would spread much opposition to such work. In fact, they would fight it on every ground, both real and imaginary. Yet in so doing they would simply display their ignorance, for notice the actual results which have been obtained from time to time in many different localities by the control of dogs—especially so in Berlin and Vienna, where rabies has been prevalent in direct proportion to the freedom given to dogs; in England, where the disease has been practically eradicated through the muzzling of dogs and destruction of all supposed cases; in Australia, where it has never appeared, due principally to the strict quarantine against all dogs seeking admission to that country.

The measures to be adopted should be both general and special—general as pertaining to all dogs, and special in those cases where the symptoms have actually appeared or where an animal has been exposed.

As general measures I would advise the licensing of all dogs; the establishing of dog wardens, whose duty it would be to enforce the dog license and to catch and destroy all dogs which have not been taken care of by their owners. In order to identify licensed dogs they should be properly provided with a tag. The warden might act in conjunction with the board of health, and so take direct charge of rabid and exposed dogs. Dogs should be excluded from the public highways except when properly muzzled and led by a chain.

Under special regulations would come the reporting of all cases of rabies to the proper authorities, the disposal of such cases, and the proper control of all exposed animals. Exposed dogs might be immediately destroyed, as a quarantine of such dogs for three or six months would not positively include every case, the period of incubation being very irregular, it frequently extending over six months. To make the period one year would not even catch every case, and as dogs live but comparatively a few years, it would be a matter of economy to destroy the dog rather than extend the quarantine period and remain in doubt for that length of time. The report of a case should be followed by a close observation of all dogs in that section for a definite period of time, say six months. These measures can be modified by the authorities according to their judgment and the observation of new cases.

In this section and west of here I wish to particularly call your attention to the possibility of wild animals conveying the disease, especially coyotes and wolves. They are quite dog-like in their nature, and act very similar to dogs when affected. They will become quite bold and venture into the roads, pastures, and farm-yards, and there attack the farm animals. In this case I should advise active measures which would annihilate them. Again, I would warn against strange dogs that are running aimlessly about the country, dirty, and perhaps having a tendency to attack objects; or to familiar dogs that are suddenly manifesting a changed disposition, as that might often be but the initial stage of rabies. The saliva being virulent several days before any noticeable change is seen, it would be advisable to discontinue the habit of allowing dogs to lick one's person.

